# Anaclitic-sociotropic and introjective-autonomic personality dimensions and depressive symptoms: a systematic review

**DOI:** 10.1186/s12991-021-00373-z

**Published:** 2021-12-16

**Authors:** Angelica Marfoli, Federica Viglia, Micaela Di Consiglio, Sheila Merola, Stefano Sdoia, Alessandro Couyoumdjian

**Affiliations:** Dipartimento di Psicologia, Sapienza Università di Roma, Via dei Marsi 78, 00185 Roma, Italy

**Keywords:** Sociotropic personality, Autonomic personality, Psychological dimensions, Depression, Depressive symptoms, Complicated grief

## Abstract

Sociotropy (anaclitic) and autonomy (introjective) are conceptualised as two personality dimensions that confer vulnerability to depression. According to Blatt and Beck’s theories, sociotropic individuals exhibit distinctive patterns of symptoms such as prominent anxiety, depressed mood, helplessness, crying and somatic concerns, while self-critical ones seem to exhibit a pattern of symptoms including prominent guilt, hopelessness, feelings of failure and worthlessness and other cognitive symptoms.

This systematic review was performed with the aim of investigating whether and to what extent psychological dimensions of anaclitic-sociotropic and introjective-autonomy are related to a specific core of depressive symptoms. The search was conducted in three databases (PubMed, PsycINFO and Scopus) and 27 articles were selected.

Results showed a weak association between somatic symptoms and dependent personality traits, while the relationship between self-criticism and cognitive symptomatology was significantly higher. These findings are discussed in the context of future research, necessary to corroborate the existence of a form of depression characterised by somatic features usually ignored by diagnostic criteria, essential to direct psychological treatments to these depressive personality differences.

## Introduction

Depression is one of the most common and invalidating mental disorders in current society [45] and it can be very heterogeneous due to several possible combinations of symptoms [39]. Zimmermann and colleagues (2015 identified 227 possible depressive patterns, suggesting that depressed people may have clinical conditions that differ drastically. Furthermore, the comorbidity of depression with other psychological or medical disorders, such as anxiety disorders or post-traumatic stress disorder (PTSD, [2, 20, 46], or even other chronic illnesses [53], shows how depressive symptomatology can vary. Also, specific behaviours that are often considered to be the clinical manifestation of major depressive disorder, particularly suicidality and suicidal ideation, seem to not be typical of depression, but can emerge from human sadness. This lack of information can also affect therapeutic efficiency [66]. The tendency to ignore symptomatic variations of Major Depressive Disorder could explain the lack of progress about the validation of under-diagnosis and the identification of differential treatments that are effective and adequate. In line with this hypothesis, Sidney Blatt [12]—from a psychoanalytic perspective—and Aaron T. Beck [8]—from a cognitive point of view—assumed that different traumatic experiences in childhood can lead to two different personality dimensions that are prone to depression in adulthood. The personality dimensions described by Blatt—anaclitic and introjective—and those described by Beck—sociotropic and autonomic—can be considered as equivalents: anaclitic and sociotropic are overlapping concepts, as are introjective and autonomic. They are also referred to as dependency and self-criticism, respectively. The anaclitic-sociotropic dimension refers to a dependent personality style that is sensitive to the disruption of interpersonal relationships and is characterised by a strong need to be loved and taken care of, together with exaggerated fears of loss and abandonment, and a tendency to seek help and support from the others, especially when faced with stress. It includes feelings of loneliness, weakness and helplessness, and it is more frequent in women. An introjective-autonomic personality instead implies a strong emphasis on control, self-definition, autonomy, and concerns about personal goals and high standards. The main feelings associated with this are self-devaluation, low self-worth, self-criticism, sense of inferiority and guilt, and it has to do with a narcissistic vision of oneself [12, 51]. Several studies [19, 30, 47, 69] in recent decades have focused on these distinctive patterns of symptoms shown by these different personality configurations according to the Symptoms Specificity Hypothesis [13], the aim of which was to clarify the specific relationships between a pre-existing depressive personality and specific depression symptoms. According to this hypothesis, sociotropic individuals in particular should show distinctive patterns of symptoms such as prominent anxiety, depressed mood, helplessness, crying and somatic concerns. Self-critical individuals, on the other hand, are more prone to developing a pattern of symptoms that includes prominent guilt, hopelessness, feelings of failure and worthlessness, suicidality, and other cognitive symptoms.

The general purpose of this systematic review is to provide robust data concerning the relationship between anaclitic-sociotropic and introjective-autonomy dimensions and specific depressive symptoms among depressed patients. Thus, different typical depressive symptoms such as anhedonia, shame, uncontrolled crying, suicidality, anger, insomnia, rumination, and self-criticism are taken into consideration to evaluate if they are more often frequent in people showing an anaclitic-sociotropic personality or an introjective-autonomic one.

The ultimate goal of this work is to demonstrate how often diagnostic criteria for depression, for example *Diagnostic and Statistical Manual of Mental Disorders* diagnostic criteria [2] tend to mainly highlight only a cognitive symptom pattern, which is typical of the introjective-autonomic configuration of depressive personality. However, little attention is paid to other depressive forms distinguished by a more somatic symptomatology, which is recurrent in anaclitic-sociotropic personalities. This manifestation also appears to be strongly related to the emotional experiences of people who suffer from Complicated Grief Disorder.

For this reason, complicated grief symptomatology will be considered in order to underline its correlation to anaclitic-sociotropic depression symptoms and to show that there are many different forms of major depressive disorder that should not be ignored.

Considering these personality differences while orienting depression treatment is another important purpose of this study.

## Method

**Fig. 1:**
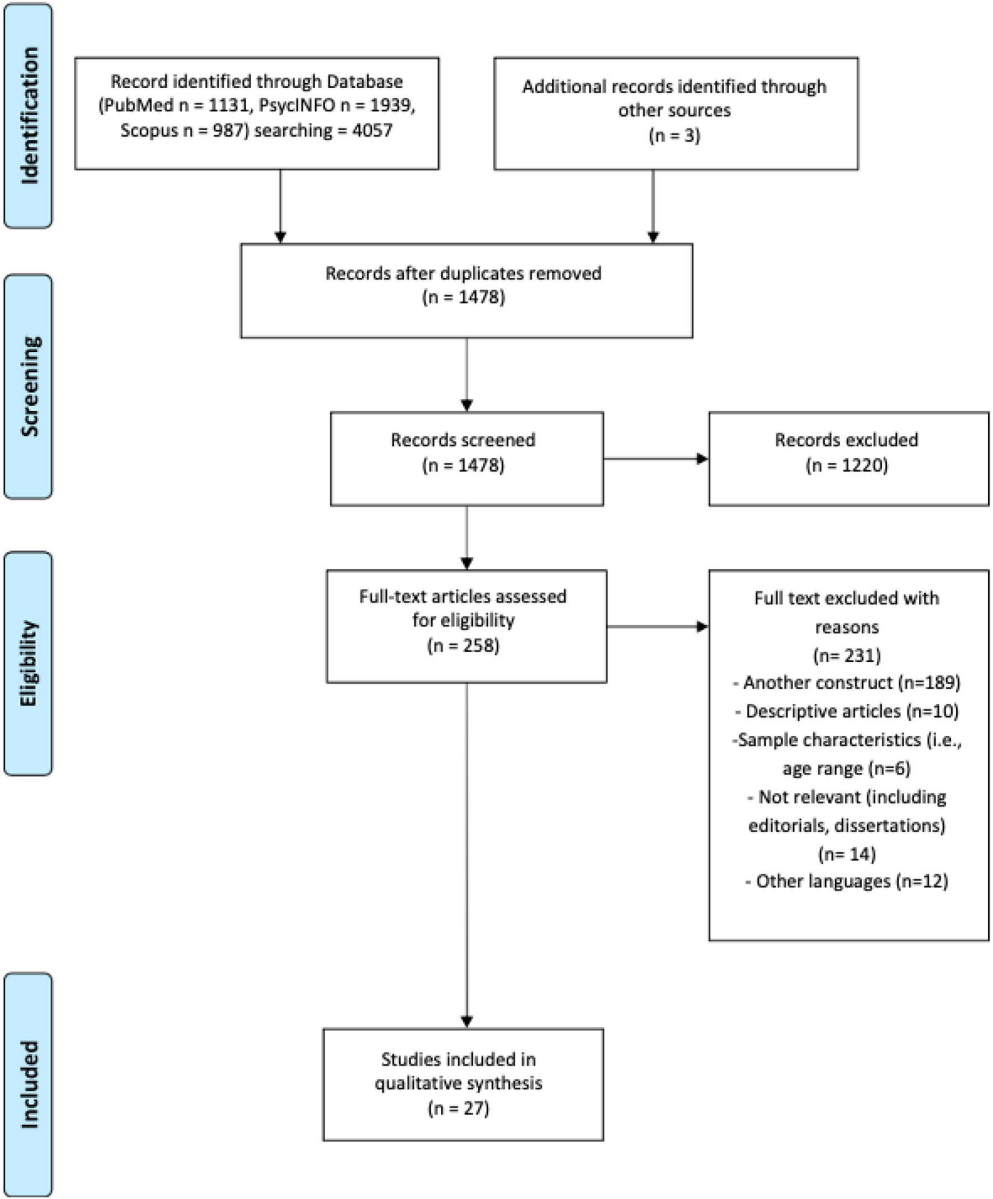
PRISMA2020 flow diagram for new systematic reviews

A systematic review was performed in compliance with the PRISMA guidelines for systematic reviews and meta-analyses (see Fig. 1) [54].

### Information sources and database search

In order to systematically collect empirical studies on the relation between personality dimensions (anaclitic-sociotropic trait versus introjective-autonomic trait) and different depressive manifestations, several keywords were used to search for appropriate publications in three electronic databases: PubMed, Scopus, PsycINFO.

Two separate reviewers conducted the search in each database for the following two groups of keywords: (a) terms related to personality traits: “anaclitic” and “introjective” personality OR trait*, “dependency” and “self-critical” personality OR trait*, “sociotropic” and “autonomic” personality OR trait*, “Depressive Experiences Questionnaire” (DEQ), “Sociotropy and Autonomy Scale” (SAS); (b) terms related to pathological outcomes: “depressive symptom*”, “depression”, “complicated grief”, “bereavement”.

Key words should be part of the title or the abstract of the literature.

### Literature search strategy and eligibility criteria

All duplicates and non-relevant records focusing on title and abstract were removed and the most relevant full texts were analysed and included according to eligibility criteria. The inclusion criteria are the following: (1) only articles published in English in peer-reviewed journals; (2) studies had to consider the relationship between anaclitic-sociotropic or introjective-autonomic personality and depressive symptoms; (3) depressive symptoms had to be assessed using a validated method; all tests measuring self-critical and dependent personality traits and depressive symptoms were selected; (4) the population group of interest were adults (over 18 years of age). Exclusion criteria are as follow: (1) the presence of comorbidities with other psychiatric disorders; (2) children’s samples.

Additionally, citations in retrieved articles were screened to identify extra relevant publications. All worthwhile articles were selected and screened based on the aforementioned eligibility criteria.

### Data extraction

The analysis was conducted by two separate reviewers, who applied the eligibility criteria in each database. The same two authors carried out the selection of the studies, separately and together. In case of disagreement on the inclusion of a study, the two authors discussed their point of view until a consensus was reached. Where necessary, a third reviewer was involved to reach a consensus.

### Assessing the quality of selected studies

The evaluation of the risk of bias was conducted by a quality index derived from the Qualsyst’ Tool [48]. The quality assessment of the studies appears from moderate to strong (see Appendix A).

## Results

**Fig. 2:**
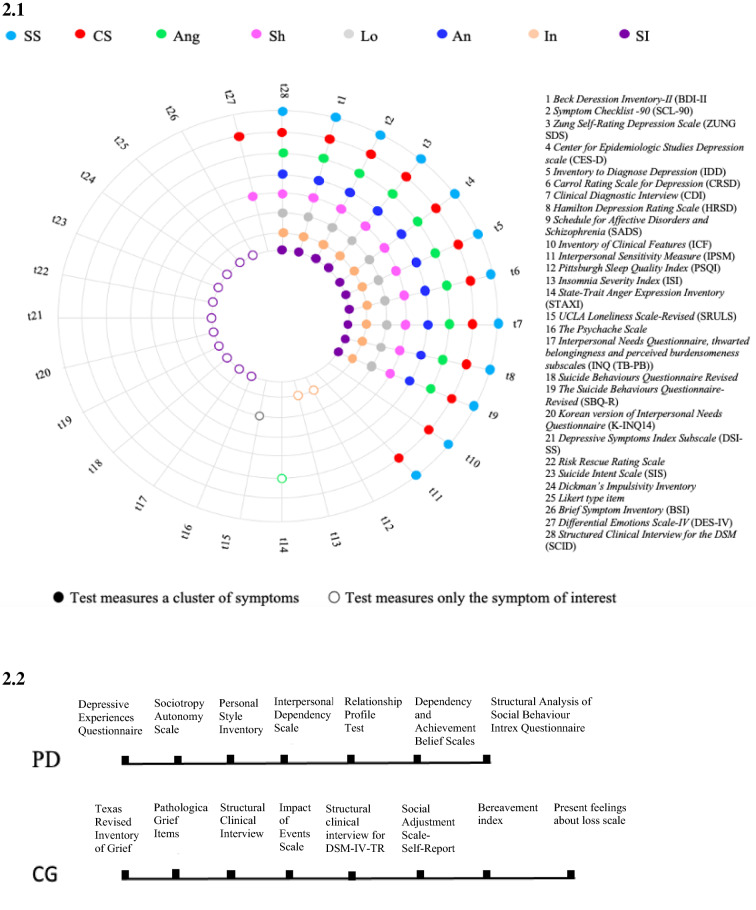
Tests measuring depressive symptoms, complicated grief, and personality dimensions. **2. 1 ***SS (Somatic Symptoms):* loss of pleasure, crying, agitation, loss of interest, loss of energy, changes in sleeping, irritability, changes in appetite, concentration difficulty, tiredness or fatigue, loss of interest in sex, interpersonal sensitivity. *CG (Cognitive Symptoms):* such as sadness, pessimism, past failure, guilty feelings, punishment feelings, self-dislike, self-criticalness, indecisiveness, worthlessness, self-blame, work difficulty, social withdrawal, hopelessness. *Ang*: Anger; *Sh*: Shame; *Lo*: Loneliness; *An*: Anhedonia; *In*: Insomnia; *SI*: Suicidal Ideation. Coloured circles indicate that the scale measures a cluster of symptoms. Empty circles indicate that the scale measures only the symptom of interest). **2. 2** Tests measuring personality dimensions and complicated grief. *PD* (Personality dimensions); *CG* (complicated Grief)

For the purpose of this systematic review, 27 studies examining the relationship between personality and depression symptoms in adults were identified, fulfilling the inclusion and exclusion criteria. Tables 1, 2 and 3 summarise data about samples, assessment of personality and depression symptoms, and the main results of each study per cluster of symptoms (somatic and cognitive symptoms, other symptoms and complicated grief, respectively). Figure 2 provides an overview of the tests that each study utilised to assess personality (Fig. 2, 2.2) and depression symptoms (Fig. 2, 2.1).

**Table 1:** Extracted data from included studies on somatic and cognitive symptoms

Reference	Country	Sample	Symptoms	Measures of Self-criticism and Dependency	Measure of psychopathology	Analysis	Results
Klein et al. [47]	–	63 outpatients 100% female 15 volunteers (control group 100% female	Pervasive anhedonia Loss of interest Decreased energy Insomnia Hypersomnia Loss of weight or appetite Increased weight or appetite Difficulty concentrating or making decisions Guilt Feelings of inadequacy or worthlessness Psychomotor retardation Psychomotor agitation Suicidal thoughts or behaviour Qualitative difference in mood Lack of reactivity of mood Diurnal variation (a.m. worse) Crying or tearfulness Social withdrawal Dependency Irritability Brooding Self-pity Somatic complaints Pessimism or hopelessness	DEQ	BDI-R CRSD	Discriminant analysis (alpha sets at 0.01)	Self-criticism Loss of interest (overall Rao’s *V* = 11.17, change in *V* = 9.50, *p* = 0.002) Irritability (overall Rao’s *V* = 6.17 change in *V* = 6.07, *p* = 0.01) Dependency Crying or tearfulness (overall Rao’s *V* = 22.69, change in *K* = 11.78, *p* < 0.001) Presence of both dependency and self-criticism Decreased energy (overall Rao’s *V* = 32.95, change in *V* = 31.99, *p* < 0.001)
Robins and Luten [69]	USA	50 depressed sample 26% male (*n* = 13) 74% female (*n* = 37) Mean age = 44.12 SD = ± 11.80	Crying Variability of mood Reactivity of mood Feeling lonely Loss of interest or pleasure Loss of interest in people Self-blame Irritability Concern about inability to function	PSI	ICF	Exploratory analysis	Sociotropy Crying Mood-variability and reactivity Loneliness Autonomy Loss of interest or pleasure Loss of interest in people Self-blame, irritability, concern about inability to function
Robins et al. [71]	Ontario	103 patients 38 men 65 females Mean age = 39.8 SD = 11.1	Theoretical sociotropic symptoms composite: Sad feelings Crying Decision-making difficulty Negative body image Somatization Depressed mood General somatic problems Somatic anxiety Positive psychic anxiety Anxiety and phobic anxiety Theoretical autonomous symptoms composite: Hopelessness Guilt Self-blame Feeling like a failure Punishment Irritability Loss of satisfaction Disappointment in self Loss of functioning Feelings of guilt Difficulty working Social withdrawal Self-blame Hopelessness Loss of interest Worthlessness Feeling critical of others	PSI	BDI HRSD SCL-90 SCID-I	Correlational analysis Exploratory analysis	Stronger correlation between autonomy and autonomous symptoms than with theoretically sociotropic symptoms (*z* = 3.03, *p* < 0.01) Sociotropy did not show the predicted pattern Sociotropy was strongly and significantly related to the sociotropic symptoms; instead, autonomy showed the opposite pattern
Desmet et al. [30]	Belgium	163 outpatients 28.22% male (*n* = 46) 71.77% female (*n* = 117) Age range 19–64 years, *M* = 39.45 SD = ± 9.97	Sadness Pessimism Past failure Guilty feelings Punishment feelings Self-dislike Self-criticalness Suicidal thoughts Crying Agitation Loss of interest Indecisiveness Worthlessness Loss of energy Changes in sleeping Irritability Changes in appetite Concentration difficulty Tiredness or fatigue Loss of interest in sex	DEQ dependency (DEP) and self-criticism (SC) subscales	BDI-II somatic and cognitive subscales	Regression analysis Conservative significance test (*p* < 0.01) F-test	*Raw items score* DEQ-DEP: Indecisiveness [*ß* = 0.229; F (1,25) = 8.811; *p* = 0.003] Worthlessness [*ß* = 0.251; F (1,25) = 12.280; *p* = 0.001] DEQ-SC: Pessimism [*ß* = 0.215; F (1,25) = 7.551; *p* = 0.007] Past failure [*ß* = 0.324; F (1,25) = 19.123; *p* = 0.000] Guilty feelings [*ß* = 0.356; F (1,25) = 23.325; *p* = 0.000] Self-dislike [*ß* = 0.390; F (1,25) = 29.484; *p* = 0.000] Self-criticalness [*ß* = 0.391; F (1,25) = 28.675; *p* = 0.000] Crying [*ß* = 0.240; F (1,25) = 9.772; *p* = 0.002] Indecisiveness [*ß* = 0.234; F (1,25) = 9.561; *p* = 0.002] Worthlessness [*ß* = 0.396; F (1,25) = 31.599; *p* = 0.000] *After ipsatization* DEQ-DEP: Worthlessness [*ß* = 0.008; F (1,155) = 8.849; *p* = 0.003] DEQ-SC: Self-dislike [*ß* = 0.390; F (1,155) = 8.228; *p* = 0.005] Self-criticalness [*ß* = 0.391; F (1,155) = 10.219; *p* = 0.002] Worthlessness [*ß* = 0.396; F (1,155) = 12.086; *p* = 0.001]
Luyten et al. [51]	Belgium	93 depressed sample 27 males 66 females Mean age = 39.24 SD = 9.46	Dependent symptom composites (D-COM): Sad mood Crying spells Feeling ugly Worrying about physical Problems Constipation Tachycardia Crying easily Feeling lonely Worrying too much about things Feeling hurt and rejected Self-critical symptom composites (SC-COM): Pessimism Feelings of failure Lack of satisfaction Guilty feelings Sense of punishment Self-hatred Self-blame Irritability Social withdrawal Indecisiveness Work inhibition Personal devaluation Feeling easily annoyed or irritated Feeling of being caught or trapped Feeling blocked in getting things done	DEQ	BDI Zung SDS SCL-90 (Depression subscale	Bivariate correlation Partial correlation	Dependency didn’t show a strong relation with the dependent composite in (Hotelling’s t(90) = 1.90, ns) Self-criticism showed a strong relation to self-critical composite (Hotelling’s t(90) =− 3.49, Ps < 0.01) Controlling for the self-critical symptom composite, dependency resulted more strongly related to the dependent symptom composite (Ps < 0.001)
Otani et al. [59]	Japan	362 healthy volunteers 58.02% male (*n* = 210) 41.98% female (*n* = 152) Mean age = 31.6 SD = ± 9.8	Interpersonal sensitivity	SAS	IPSM total and Interpersonal Awareness, Separation anxiety, Timidity and Fragile Inner Self subscales	Linear regression analysis and multiple regression analysis	Authors considered a p-value less than 0.05 statistically significant *Correlational analysis among IPSM and SAS scores* Sociotropy IPSM total (*r* = 0.621; *p* < 0.001) Interpersonal awareness (*r* = 0.551; *p* < 0.001) Separation anxiety (*r* = 0.569; *p* < 0.001) Timidity (*r* = 0.513; *p* < 0.001) Fragile inner self (*r* = 0.419; *p* < 0.001) Autonomy IPSM total (*r* = 0.152; *p* < 0.01) Interpersonal awareness (*r* = 0.114; *p* < 0.05) Separation anxiety (*r* = 0.160; *p* < 0.01) Fragile inner self (*r* = 0.193; *p* < 0.001) *Multiple regression* Sociotropy IPSM total (*ß* = 0.613; *p* < 0.001) Interpersonal Awareness (*ß* = 0.547; *p* < 0.001) Separation Anxiety (*ß* = 0.558; *p* < 0.001) Timidity (*ß* = 0.518; *p* < 0.001) Fragile Inner Self (*ß* = 0. 0.398; *p* < 0.001) Autonomy Fragile Inner Self (*ß* = 0.130; *p* < 0.01)
Straccamore et al. [79]	Italy	51 outpatients 33.33% male (*n* = 17) 66.66% female (*n* = 34) Mean age = 51.59 SD = ± 11.68	Sadness Pessimism Past failure Loss of pleasure Guilty feelings Punishment feelings Self-dislike Self-criticalness Suicidal thoughts Crying Agitation Loss of interest Indecisiveness Worthlessness Loss of energy Changes in sleeping Irritability Changes in appetite Concentration difficulty Tiredness or fatigue Loss of interest in sex Depressed mood Anxiety psychic Anxiety somatic Retardation Depersonalization and derealization	DEQ	CDI HAMD BDI-II	Regression analysis	*Relation between DEQ personality factors and BDI-II symptoms* Self-criticism Pessimism (*β* = 0.379, *t* = 2.869, *p* = 0.006) Past Failure (*β* = 0.436, *t* = 3.391, *p* = 0.001) Guilty Feelings (*β* = 0.406, *t* = 3.112, *p* = 0.003) Punishment Feelings (*β* = 0.341, *t* = 2.540, *p* = 0.014) Self-dislike (*β* = 0.392, *t* = 2.987, *p* = 0.004) Self-criticalness (*β* = 0.437, *t* = 3.400, *p* = 0.001) Loss of interest (*β* = 0.328, *t* = 2.430, *p* = 0.019) Indecisiveness (*β* = 0.306, *t* = 2.248, *p* = 0.029) Change in Appetite (*β* = 0.363, *t* = 2.723, *p* = 0.009) Concentration Difficulty (*β* = 0.299, *t* = 2.194, *p* = 0.033) Tiredness or Fatigue (*β* = 0,321, *t* = 2.375, *p* = 0.021) Dependency Guilty feelings (*β* = 0.383, *t* = 2.898, *p* = 0.006) *Relation between DEQ personality factors and HAMD symptoms* Self-criticism Depressed Mood (*β* = 0.396, *t* = 3.022, *p* = 0.004) Anxiety Psychic (*β* = 0.294, *t* = 2.157, *p* = 0.036) Anxiety Somatic (*β* = 0.336, *t* = 2.496, *p* = 0.016) Dependency Retardation (*β* = 0.298, *t* = 2.188, *p* = 0.033) Anxiety Psychic (*β* = 0.321, *t* = 2.373, *p* = 0.022) Depersonalization and Derealization (*β* = − 0.358, *t* = − 2.685, *p* = 0.010)

**Table 2:** Extracted data from included studies about other depressive symptoms (Loneliness, Shame, Guilt, Embarrassment, Interpersonal intimacy, Self-punitiveness, Anhedonia, Hopelessness, Anger, Insomnia, Suicidality)

Reference	Country	Sample	Symptoms	Measures of self-criticism and dependency	Measures of psychopathology	Analysis	Results
Schachter and Zlotogorski [74]	Israel	58 volunteers 35 males 35 females	Loneliness	DEQ	SRULS	Regression analysis Inter-correlational analysis	Self- criticism (*β* = 0.63, *t* = 6.761, *p* < 0.05) Dependency (*β* = 0.29, *t* = 2.888, *p* < 0.05) Dependency (*r* = 0.34, *p* < 0.01) Self-criticism (*r* = 0.67, *p* < 0.01)
Burke and Haslam, [19]	USA	74 depressed patients 39 females (53%) 35 males (47%) Age range: 19- 61 years Mean age = 39.0	Self-punitiveness (guilt, feelings of failure), Anhedonic symptoms (loss of interest, fatigue), Hopelessness (pessimism, suicidal ideation)	SAS, PSI-R, DEQ, DABS	BDI, IDD	Principal components analysis, Correlation analyses	Correlation analyses: - *Self-direction and freedom from attachments component* of *autonomy* (*core autonomy*) and anhedonic symptoms: *r* = 30, *p* < 0.01 - *Concern with others’ disapproval component* of *dependency* and self-punitive symptoms: *r* = 34, *p* < 0.01 - *Self-criticism/perfectionism component* of *autonomy* and self-punitive symptoms: *r* = 57, *p* < 0.001 - *Self-criticism/perfectionism component* of *autonomy* and hopelessness: *r* = 32, *p* < 0.01
Besser et al. [11]	Canada	167 volunteers 86 males 81 females Mean age = 21.61 SD = 4.07	Loneliness	DEQ (McGill revision)	CES-D UCLA Loneliness Scale-Revised	Zero-order correlations Regression analysis	*Self-criticism* (*r* = 0.43, *p* < 0.0001 and *r* = 0.62, *p* < 0.0001 for intimate and non-intimate relationships, respectively) *Dependency* (*r* = 0.22, *p* < 0.05 and *r* = 0.13, *p* = 0.22 for intimate and non-intimate relationships, respectively) *CES-D* (*ß* = 0.44, *t* = 3.94, *p* < 0.0001 and *ß* = 0.24, *t* = 2.18, *p* < 0.03 for relationship and no relationship subsamples, respectively) *Self-criticism* (*ß* = 0.22, *t* = 2.05, *p* < 0.04 and *ß* = 0.46, *t* = 4.28, *p* < 0.0001 for the relationship and no relationship subsamples, respectively) The low effect of dependency on loneliness found in the zero-order correlations for the romantic relationships group was no longer evident when controlling for participants’ levels of depressive symptoms (*ß* = 0.04, *t* = 0.43, *p* = 0.18 and *ß* = 0.05, *t* = 0.65, *p* = 0.5\2 for relationship and no relationship subsamples, respectively)
Fazaa and Page [36]	Canada	807 university students Caucasian (76%); the remaining 24% came from the Middle East, Africa, the Caribbean, or Asia Mean age: 20 years	Suicidality	DEQ (66 items)	BDI-II Risk Rescue Rating Scale (lethality of attempts)	Correlations Standard multiple regressions	Self-criticism-risk (*r* = 0.53), Self-criticism-risk rescue (*r* = 0.55) Self-criticism subjective lethality (*r* = 0.42) Self-criticism-intent score (*r* = 0.49) Self-criticism-rescue (*r* = − 0.50) Self-criticism- subjective lethality (*β* = 0.50) Self-criticism-intensity of wish to die (*β* = 0.76) Dependency-subjective lethality (*β* = − 1.57) Dependency-intensity of wish to die (*β* = − 1.83)
Vanhuele et al. [80]	Belgium	134 adult outpatients (DMS-IV mild-severe Depression)	Suicidality	DEQ (66 items)	BDI-II	Latent class analysis	Self-directed aggression: self-mutilation -suicide attempts self-critical
Fazaa and Page [37]	Canada	96 students (13 male, 83 female) 75% Caucasian, remaining sample from Middle Eastern, African, Asian, and Hispanic individuals	Suicidality (Impulsivity, intent, and lethality)	DEQ (66 items)	SIS (Suicide intent with previous attempt) (2 items) Dickman’s Impulsivity Inventory (23 items self-report) Likert type item (Suicide item) Risk Rescue Rating Scale (Lethality of attempts) (10 items)	Discriminant Function Analysis (DFA) Receiver Operating characteristics curve (ROC) analysis	Correlation Dependency-State impulsivity *r* = 0.40, *n* = 96, *p* < 0.01) Correlation Self- criticism- State Impulsivity (*r* = 0.35, *n* = 96, *p* < 0.01)
O’ Riley and Fiske [57]	USA	636 adults (70.9% women; 92.2% European American) Age range: 18–24 years	Suicidality	PSI-II	SBQ-14 (propensity for suicidal behaviour) (14 items)	Pearson’s correlation (relationship between autonomy and propensity for suicidality) Two multiple linear regressions (association between propensity for suicidality and autonomy subscales)	Young sample: Autonomy- suicidality (*r* = 0.27) Older sample: Need for Control-suicidality (*r* = 0.26) Defensive Separation (*β* = 0.11, SE = 0.03, *p* < 0.01, 95% CI = 0.05, 0.16), Perfectionism (*β* = 0.38, SE = 0.06, *P* < 0.01, 95% CI = 0.25, 0.50), Need for Control (*β* = − 0.05, SE = 0.04, *p* > 0.05, 95% CI = − 0.01, 0.04) and suicidality in younger sample; Need for Control (*β* = 0.21, SE = 0.08, *p* < 0.01, 95% CI = 0.05, 0.37), Defensive Separation (*β* − 0.04, SE = 0.06, *p* > 0.013, 95% CI = − 0.14, 0.07), Perfectionism (*β* = − 0.10, SE = 0.14, *p* > 0.013, 95% CI = − 0.38, 0.19) and suicidality in older sample
Campos et al. [23]	Portugal	105 volunteers adults (51 male, 54 female) Age range: 19–64 years M: 36.3 SD: 11.5	Suicidality Through distress	DEQ (66 items)	BSI (53 items self-report) Sociodemographic Questionnaires (2 items on suicidality)	Structural equation modelling (SEM)	Direct association model: Self-criticism-Suicidality: (*β* = 0.40, *t* = 2.394, *p* < 0.017) Dependency-Suicidality: (*β* = 0.10, *t* = 0.712, ns) Mediational structural equation modelling: Self-criticism-Suicidality through distress: (*β* = 0.54, *t* = 6.452, *p* < 0.0001) Dependency-Distress: (*β* = 0.36, *t* = 4.459, *p* < 0.0001) Distress-Suicidality: (*β* = 0.51, *t* = 2.284, *p* < 0.022)
Dorahy and Hanna [32]	New Zealand and Northern Ireland	315 students 17.1% males (*n* = 54) 82.9% females (*n* = 261) Age range = 18–64 years Mean age = 22.54 SD = 7.24	Shame, guilt, embarrassment Interpersonal intimacy	DEQ-SF	DES-IV, shame, guilt, and hostility-inward subscales (SG&HI-DES-IV)	Path analysis	Standardized Regression Coefficients for Each of the Model Paths (p = < 0.01): *Introjective orientation* - Embarrassment: *β* = 0.281 - Shame: *β* = 0.381 - Guilt: *β* = 0.232 - Interpersonal Intimacy: *β* = − 0.426 *Anaclitic orientation*: - Embarrassment: *β* = 0.359 - Guilt: *β* = 0.215 - Interpersonal Intimacy: *β* = 0.266
Abi-Habib and Luyten [1]	Belgium	253 community adults 58.33% females Mean age = 32.21 SD = 5.40	Anger	DEQ	BDI STAXI	Zero-order correlations	*Self-criticism* (*p* < 0.01) - State anger: *r* = 0.177 - Trait anger: *r* = 0.393 - Anger-control: *r* = − 0.220 - Anger-in: *r* = 0.455 - Anger-out: *r* = 0.319 *Dependency* - State anger: *r* = 0.045 - Trait anger: *r* = 0.060 - Anger-control: *r* = 0.070 - Anger-in: *r* = 0.119 - Anger-out: *r* = − 0.117
Campos and Holden [21]	Portugal	810 non-clinical adults Age range: 19–67 years M: 36.34 SD: 12.46	Suicidality	DEQ (66 items)	CES-D (20 items) SBQ-R (4 items)	Discriminant Function Analysis (DFA) Receiver Operating characteristics curve (ROC) analysis	Standardized discriminant function coefficient (0.46 (95% CI (0.13, 0.66)) for self-criticism
Campos and Holden [22]	Portugal	200 adults (102 men, 98 women) Age range: 19–67 years M: 36,7 years D: 12,8	Suicidality (ideation and attempt, recent ideation, intention and future probability)	DEQ (66 items)	CES-D (20 items) The Psychache Scale (13 items self-report) INQ (TB-PB) (15 items self-report) Suicide Behaviours Questionnaire-Revised (4 items)	Structural Equation Modelling (SEM)	Indirect effects Self-criticism-suicidality: (*β* = 0.20, *t* = 4.17, *p* < 0.001; SE = 0.029, 95% CI [0.11, 0.30], *p* < 0.001) Neediness-suicidality: (*β* = 0.21, *t* = 4.71, *p* < 0.001; SE = 0.028, 95% CI [0.14, 0.30], *p* < 0.001)
O’ Keefe et al. [56]	USA	113 Under-graduated students (75,3% women; 93,4% Caucasian, 5,7% African Americans, 0,9% Asian Americans) M: 19.43 DS: 2.28	Suicidality	PSI.II (48 items)	CES-D (20 items) INQ (TB-PB)	Structural Equation Modelling	Time 1 autonomy predicted Time 2 depression symptoms (*β* = 0.137, *p* = 0.002) Time 2 depression symptoms predicted Time 3 perceived burdensomeness (*β* = 0.251, *p* = 0.002) and Time 3 thwarted belongingness (*β* = 0.283, *p* = 0.005)
Silva et al. [76]	Chile	177 undergraduate students (Normal: 52 introjective: 38 anaclitic: 38 mixed AI: 49) 71 males; 106 females Mean age = 21.1 SD = 1.65	Anhedonia	DEQ	BDI (anhedonia and melancholia subscales)	One-way ANOVA *post-hoc* comparisons	Anhedonia: group effect [*F* (3, 176) = 5.64, *p* < 0.01, *η*2 = 0.08] normal vs. introjective (ΔM = − 1.20, *SE* = 0.32, *p* < 0.01 Bonferroni) normal vs. mixed AI (Δ*M* = –0.98, *SE* = 0.30, *p* < 0.01 Bonferroni)
Park and Kim [60, 61]	South Korea	334 students (113 male, 200 female) Age range: 19–27 years M: 21.51 DS: 1.95	Suicidality	Personal Style Inventory-II (Korean version; 18–19 items	K-INQ14) (14 items) (DSI-SS) (4 items) K-DBI-II (21 items)	Correlations Hierarchical regression	Sociotropy—BDI-II: *r* (311) = 0.23, *p* < 0.001 Autonomy-BDI-II: *r* (311) = 0.25, *p* < 0.00 Autonomy-Suicide ideation: *r* (311) = 0.16, *p* < 0.01 Model 2: PB (*β* = 0.24, *t* (298) = 0.42, *p* < 0.001) and sociotropic personality, *β* = − 0.11, *t* (298) = − 2.26, *p* < 0.05 predicted suicide Model 3: Significance TB and sociotropy (*β* = − 0.11, *t* (294) = − 2.01, *p* < 0.05) and PB and autonomy (*β* = 0.19, *t* (294) = 2.94, *p* < 0.01)
Bar et al. [4]	Israel	161 young adults 36 males 125 females Age range = 20–30 years Mean age = 25 SD = 1.4	Insomnia	DEQ-SC6	BDI-II PSQI ISI	Regression analysis	Association between self-criticism (Time 1) and insomnia (Time 2) evidenced a trend (*β* = 0.12, SE = 0.07, *p* = 0.09, 95% CI [− 0.02, 0.26]) Two-wave interaction: the self-criticism by depressive symptoms interaction predicted time 2 insomnia (*β* = 0.19, SE = 0.07, p = 0.007, 95% CI [0.05, 0.33]) The positive association between time 1 self-criticism and time 2 insomnia was marginally significant for individuals with high (1SD above the mean) levels of depressive symptoms (B = 0.03, SE = 0.02, *p* = 0.07, 95% CI [− 0.00, 0.08]), but not for individuals with mean (*B* = 0.01, SE = 0.01, *p* = 0.40, 95% CI [− 0.02, 0.05]) and below mean (1SD below the mean) levels of depressive symptoms (*B* = − 0.00, SE = 0.02, *p* = 0.68, 95% CI [− 0.05, 0.03]) At 2SDs above the mean of depressive symptoms, the positive association between time 1 self-criticism and time 2 insomnia was statistically significant (*B* = 0.06, SE = 0.03, *p* = 0.04, 95% CI [0.00, 0.12])

**Table 3:** Extracted data from included studies about complicated grief

Reference	Country	Sample	Symptoms	Main Personality Traits	Measure of Dependency	Measure of psychopathology	Analysis	Results
Piper et al. [65]	Canada	277 psychiatric outpatients 70% women Types of losses: parent (45%), partner (10%), sibling (9%), friend (8.4%), child (7%), grandparents (5%), other (15%) Age range M: 43,1	Grief symptoms	Patient’s promotion of dependence of the deceased	INTREX Questionnaire (16 items)	Present Feeling Subscale of the Texas Revised Inventory of Grief (TRIG) PGI IES SAS-SR	Pearson Correlations, Stepwise Regression Analyses	Pearson Correlations: Significant direct association between patient’s *promotion of dependence of the deceased* and TRIG grief score: r [129] = 0.21, *p* = 0.015 Stepwise Regression Analysis: *Patient’s promotion of dependence of the deceased* accounted for 4% of the variation in the TRIG grief score
Bonanno et al. [16]	USA	205 widowed persons (non- clinical sample) 180 male, 25 female Age range M: 72 SD: 6.5	Grief symptoms	Interpersonal Dependency, Dependency on the spouse	Interpersonal Dependency Scale (5 items)	Bereavement index Present feelings about loss scale Texas Revised Inventory of Grief (TRIG)	ANOVA	*Interpersonal Dependency* (F (4–80) = 3.30, *p* < 0.05): - Chronic grievers: *M* = 0.31, SD = 0.88 - Resilient individuals: *M* = 0.11, SD = 0.89 *Dependency on the spouse* (F (4–80) = 2.58, *p* < 0.05): - Chronic grievers: *M* = 0.19, SD = 0.86 - Resilient individuals: *M* = 0.29, SD = 1.10
Denckla et al. [28]	USA	102 non-clinical sample (Married 36, prolonged grief 25, resolved grief 41)	Grief symptoms	Healthy Dependency, Destructive Overdependence	RPT	Structured Clinical Interview for DSM-IV-TR Axis I Disorders	ANOVA	*Healthy Dependency* (F = 5.12, *p* = 0.008): - Prolonged: *M* = 31.00, SD = 6.52 - Resolved: *M* = 35.73, SD = 6.44 *Destructive Overdependence* (F = 0.12, *p* = 0.883): - Prolonged: *M* = 27.36, SD = 9.39 - Resolved: *M* = 26.22, SD = 8.46
Mancini et al. [52]	USA	178 non-clinical sample (104 bereaved, 74 married participants; 33 resilient, 40 recovered, 31 prolonged grievers) Age: under 65 Bereaved: M: 51.43, SD: 9.48 Married M: 49.42, SD: 9.37	Grief symptoms	Healthy Dependency, Destructive Overdependence	RPT	Structured clinical interview	Univariate analyses, multivariate analyses (polychotomous logistic regression model)	Univariate analysis *Healthy Dependency* (F = 5.16, *p* < 0.05): - Prolonged: *M* = 3.19, SD = 0.66 - Resilient: *M* = 3.65, SD = 0.55 - Recovered: *M* = 3.66, SD = 0.54 *Destructive Overdependence* (*F* = 1.22, *p* < 0.25): - Prolonged: *M* = 2.85, SD = 0.83 - Resilient: *M* = 2.43, SD = 0.71 Multivariate analysis: *Healthy Dependency*: - Prolonged vs. resilient: OR [95% CI] = 2.35 [0.25, 0.22, 0.06] - Recovered vs. Prolonged: OR [95% CI] = 5.98 [1.26, 28.35] *Destructive Overdependence:* - Prolonged vs. resilient: OR [95% CI] = 6.42 [1.70, 24.21] - Recovered vs. prolonged: OR [95% CI] = 0.51 [0.22, 1.18]

In the following paragraphs, all included articles have been described according to the cluster of symptoms.

### Somatic and cognitive symptoms

Seven studies [30, 47, 51, 59, 69, 71, 79] were identified examining the relationship between personality and somatic symptoms in adults (age range 18–70 years).

The main result is a significant positive association between dependent personality and *indecisiveness* [*ß* = 0.229; F(1,25) = 8.811; *p* = 0.003], *worthlessness* [*ß* = 0.251; F(1,25) = 12.280; *p* = 0.001] [30], *and guilty feelings* (*β* = 0.383, t = 2.898, *p* = 0.006) [79]. The self-critical personality also showed significant positive associations with *Beck Depression Inventory-II* [9] symptoms: *pessimism* [*ß* = 0.215; F(1,25) = 7.551; *p* = 0.007] [30], (β = 0.379, t = 2.869, *p* = 0.006) [79], *past failure* [β = 0.324; F(1,25) = 19.123; *p* = 0.000] [30], (*β* = 0.436, *t* = 3.391, *p* = 0.001) [79], *guilty feelings* [*ß* = 0.356; F(1,25) = 23.325; *p* = 0.000] [30], (*β* = 0.406, *t* = 3.112, *p* = 0.003) [79], *self-dislike* [*ß* = 0.390; F(1,25) = 29.484; *p* = 0.000][30], (*β* = 0.392, *t* = 2.987, *p* = 0.004) [79], *self-criticalness* [*ß* = 0.391; F(1,25) = 28.675; *p* = 0.000] [30], (*β* = 0.437, *t* = 3.400, *p* = 0.001) [79], *crying* [*ß* = 0.240; F(1,25) = 9.772; *p* = 0.002] [30], *indecisiveness* [*ß* = 0.234; F(1,25) = 9.561; *p* = 0.002] [30], (*β* = 0.306, *t* = 2.248, *p* = 0.029) [79], *worthlessness* [*ß* = 0.396; F(1,25) = 31.599; *p* = 0.000] [30], *punishment feelings* (β = 0.341, t = 2.540, *p* = 0.014) [79], *loss of interest* (*β* = 0.328, *t* = 2.430, *p* = 0.019) [79], *change in appetite* (*β* = 0.363, *t* = 2.723, *p* = 0.009) [79], *difficulty concentrating* (*β* = 0.299, *t* = 2.194, *p* = 0.033) [79], and *tiredness or fatigue* (*β* = 0,321, *t* = 2.375, *p* = 0.021) [79].

Results also show a significant relationship between Sociotropy and *mood-variability, reactivity* and *loneliness*, as well as Autonomy and *loss of interest or pleasure, loss of interest in people, self-blame, irritability,* and *concern about inability to function *[69], similar to Klein and colleagues’ study [47] reporting higher levels of self-criticism being associated with the presence of *loss of interest* (overall Rao’s *V* = 11.17, change in *V* = 9.50, *p* = 0.002), and *irritability* (overall Rao’s *V* = 6.17 change in *V* = 6.07, *p* = 0.01). Furthermore, in the same study, higher levels of dependency were significantly associated with the presence of only one symptom, such as *crying or tearfulness* (overall Rao’s *V* = 22.69, change in *K* = 11.78, *p* < 0.001). [47].

Considering a theoretical sociotropic and autonomous symptoms composite as the sum of standardised scores on Beck Depression Inventory (BDI, [6] items, *Hamilton Rating Scale for Depression* (HRSD, [40] items and the *Symptom Checklist-90* (SCL-90; [3] items, Robins and colleagues [71] report a stronger correlation between autonomy and autonomous symptoms (BDI items—*hopelessness, guilt, self-blame, feeling like a failure, punishment, irritability, loss of satisfaction, disappointment in self* and *loss of functioning*; HRSD items—*feelings of guilt, difficulty working* and *social withdrawal*; SCL-90 items—*self-blame, hopelessness, loss of interest, worthlessness* and *feeling critical of others)* than with theoretically sociotropic symptoms (BDI items—*sad feelings, crying, decision-making difficulty, negative body image* and *somatization;* HRSD *items—*d*epressed mood, general somatic problems, somatic anxiety,* and *positive psychic anxiety; SCL-90 items—*all anxiety and phobic anxiety) (*z* = 3.03, *p* < 0.01). Instead, sociotropy does not show the predicted pattern [71].

Luyten et al. [51] also considered dependent (D-COM) and self-critical (SC-COM) symptom composites. For D-COM, the authors identified symptoms such as: *sad mood, feeling ugly, crying spells, worrying about physical problems* (BDI), *constipation, tachycardia, crying spells* (*ZUNG Self-Rating Depression Scale*; ZUNG-SDS, 1965), *crying easily, feeling lonely, worrying too much about things* and *feeling hurt and rejected* (SCL-90). For SC-COM they considered the items: *pessimism, guilty feeling, self-blame, irritability, indecisiveness, feelings of failure, work inhibition, sense of punishment, lack of satisfaction, self-hatred, social withdrawal* (BDI), *irritability, indecisiveness, personal devaluation* (ZUNG-SDS), *feeling blocked in getting things done, feeling easily annoyed or irritated* and *feeling of being caught or trapped* (SCL-90). Dependency doesn’t show a strong relationship with the dependent composite in the *Major Depressive Disorder* sample (Hotelling’s t (90) = 1.90, ns). By contrast, self-criticism shows a strong relationship with the self-critical composite in the *Major Depressive Disorder* sample (Hotelling’s t (90) = − 3.49, Ps < 0.01). To specifically examine the Interpersonal Sensitivity (*r* = 0.621; *p* < 0.001), Otani and colleagues [59] demonstrated a significant correlation between sociotropy and interpersonal sensitivity (*r* = 0.621; *p* < 0.001) and its subscales, such as *interpersonal awareness* (*r* = 0.551; *p* < 0.001), *separation anxiety* (*r* = 0.569; *p* < 0.001)*, timidity *(*r* = 0.513; *p* < 0.001)*,* and *fragile inner self* (*r* = 0.419; *p* < 0.001); whereas only the *fragile inner self* subscale was significantly correlated (*r* = 0.193; *p* < 0.001*)* with the autonomy subscale. Also, multiple regression analyses showed that sociotropy predicted total interpersonal sensitivity scores (*ß* = 0.613; *p* < 0.001), *interpersonal awareness* (*ß* = 0.547; *p* < 0.001), *separation anxiety* (*ß* = 0.558; *p* < 0.001), *timidity* (*ß* = 0.518; *p* < 0.001), *fragile inner self* (*ß* = 0. 0.398; *p* < 0.001), with autonomy predicting only *fragile inner self* (*ß* = 0.130; *p* < 0.01).

### Other depressive symptoms (loneliness, self-conscious emotions, anhedonia, insomnia, anger)

Seven studies [1, 4, 11, 19, 32, 74, 76] examined potential differences between autonomic and sociotropic personality styles in levels of loneliness, shame, guilt, embarrassment, interpersonal intimacy, self-punitiveness, anhedonia, hopelessness, insomnia, and anger.

A positive and stronger association is reported between self-criticism and loneliness (*r* = 0.43, *p* < 0.0001; *r* = 0.62, *P* < 0.0001 for self-criticism for intimate and non-intimate relationship, respectively) [11]; (*r* = 0.67, *p* < 0.01) [74] and a smaller association between dependency and loneliness (*r* = 0.22, *p* < 0.05; *r* = 0.13, *p* = 0.22 for intimate and non-intimate relationships, respectively) [11], (*r* = 0.34, *p* < 0.01) [74]. Regression analysis also revealed that self-criticism has a stronger effect in predicting loneliness (*β* = 0.22, *t* = 2.05, *p* < 0.04 and *β* = 0.46, *t* = 4.28, *p* < 0.0001, in intimate and non-intimate relationships groups, respectively) [11], (*β* = 0.63, *t* = 6.761, *p* < 0.05) [74] compared to dependency (*β* = 0.04, *t* = 0.43, *p* = 0.18 and *β* = 0.05, *t* = 0.65, *p* = 0.52, in intimate and non-intimate relationships) [11], (*β* = 0.29, *t* = 2.888, *p* < 0.05) [74].

Regarding self-conscious emotions, introjective orientation appears to be significantly associated (*p* ≤ 0.01) with increased embarrassment (*β* = 0.281), shame (*β* = 0.381), guilt (*β* = 0.232), and it is also reported to predict (*p* ≤ 0.01) significantly reduced interpersonal intimacy (*β* = − 0.426). The self-criticism subcomponent was also related to self-punitive symptoms (*r* = 57) and hopelessness (*r* = 32) [19]. By contrast, anaclitic orientation appears to be significantly associated (*p* ≤ 0.01) only with embarrassment (*β* = 0.359) and guilt (*β* = 0.215) and it significantly predicts increased interpersonal intimacy (*β* = 0.266) [32], an association between dependency and self-punitive symptoms (*r* = 34 has also been found [19]. An enhanced anhedonic symptomatology also emerged in introjective but not in anaclitic individuals compared to normal ones (normal vs. introjective ∆M =  − 1.20, SE = 0.32, *p* < 0.01 Bonferroni) [76], in line with the association between anhedonia and only “pure” autonomy components (*r* = 30) [19]. Furthermore, in Bar and colleagues’ study [4], they observed that self-criticism predicts insomnia only in individuals with quite high (2SDs above the mean depression symptoms (*β* = 0.06, SE = 0.03, *p* = 0.04, 95% CI [0.00, 0.12], and it is marginally significant for those with high (1SD above the mean depression symptoms (*β* = 0.03, SE = 0.02, *p* = 0.07, 95% CI [− 0.00, 0.08], and not significant in those with mean (*β* = 0.01, SE = 0.01, *p* = 0.40, 95% CI [− 0.02, 0.05] and below mean (1SD below the mean levels of depression symptoms (*β* =  − 0.00, SE = 0.02, *p* = 0.68, 95% CI [− 0.05, 0.03]. Finally, findings suggest that self-criticism is significantly associated (*p* < 0.01) with high levels of both state (*r* = 0.177) and trait anger (*r* = 0.393), low anger control (*r* = − 0.220), and high levels of anger towards the self (*r* = 0.455) and others (*r* = 0.319), whereas dependency appears to be related with high levels of trait anger (r = 0.060), the turning of anger towards the self (*r* = 0.119), and low levels of anger directed towards others (*r* = − 0.117) [1].

### Suicidality

Six [21, 36, 37, 57, 74, 80] examined whether the personality dimensions of self-criticism and dependency are differently associated with suicidal behaviour and the subcategories related to it. Self-critical individuals are shown to have a higher tendency toward suicide than dependents [74]. Pearson r correlations indicate a total correlation between autonomy and its subscales with suicidal ideation (*r* (311) = 0.16, *p* < 0.01.) [60, 61], with people scoring higher on self-criticism also showing higher risk (*r* = 0.53), risk-rescue (*r* = 0.55), subjective lethality (*r* = 0.42), intent scores (*r* = 0.49), and lower rescue scores (*r* = − 0.50) compared to dependents (*r* = − 0.44), (*r* = − 0.55); (*r* = − 0.25); (*r* = − 0.24); (*r* = 0.58). Furthermore, standard multiple regressions showed that only self-criticism was a significant predictor of subjective lethality (*β* = 0.50) and of the intensity of a person’s desire to die (*β* = *0.*76) while dependency was not (*β* = − 1.57); (*β* = − 1.83) [36]. Another study found standardised discriminant function coefficients for self-criticism as a predictor of suicidal behaviour to be 0.46 (95% CI (0.13, 0.66)), suggesting its implication in suicidality and general psychological distress [21]. This result was also confirmed in another study [23], with a structural equation modelling or direct association model (SEM) demonstrating a significant association only between self-criticism and suicidality (*β* = 0.40, *t* = 2.394, *p* < 0.017), and a non-significant association between dependency and suicidality (*β* = 0.10, *t* = 0.712, ns). A 2 × 2 between subject multivariate analysis of variance (MANOVA) confirmed a difference between dependency and self-criticism on different lethality indices (*p* = 0.05), and a significant negative correlation was found between dependency and state impulsivity (*r* = 0.40, *n* = 96, *p* < 0.01), while a significant positive correlation was found between self-criticism and state impulsivity (*r* = 0.35, *p* < 0.01) [37]. Moreover, a difference has been found between younger adult and older adult samples concerning the association between the autonomic personality trait and its subscales—Need for Control, Perfectionism and Defensive Separation—measured on the PSI-II [70] and suicidal behaviour [57]. In the younger adult sample, the total score on the autonomy scale (*r* = 0.27 and each autonomy subscales of Need for Control (*r* = 0.16, Perfectionism (*r* = 0.29 and Defensive Separation (*r* = 0.23 was significantly and positively associated with suicidality, while in the older adult sample, this association was only shown in Need for Control (*r* = 0.26. Also, multiple linear regressions examining the association between propensity for suicidal behaviour and autonomy subscales have indicated only Defensive Separation (*β* = 0.11, *SE* = 0.03, *p* < 0.01, 95%CI = 0.05, 0.16), and Perfectionism (*β* = 0.38, SE = 0.06, *p* < 0.01, 95% CI = 0.25, 0.50) as being significantly related to suicidality in the younger sample; as opposed to Need for Control (*β* =  − 0.05, *SE* = 0.04, *p* > 0.05, 95% CI =  − 0.01, 0.04). In contrast, in older people only Need for Control appears to be significantly and positively associated with propensity for suicidality (*β* = 0.21, *SE* = 0.08, *p* < 0.01, 95% CI = 0.05, 0.37), while Defensive Separation (*β* =  − 0.04, *SE* = 0.06, *p* > 0.013, 95% CI =  − 0.14, 0.07) and Perfectionism (*β* =  − 0.10, *SE* = 0.14, *p* > 0.013, 95% CI =  − 0.38, 0.19) are not.

### Indirect effects and distress of suicidality

In studies examining if the interaction between the independent variables of interpersonal needs—perceived burdensomeness (PB) and thwarted belongingness (TB)—and self-criticism and dependency predict suicidality or suicidal ideation dependent variables, regression analysis in a model including TB, PB, sociotropy and autonomy for moderation effects revealed TB not being a valid predictor of suicidal ideation, while PB (*β* = 0.24, *t* (298) = 0.42, *p* < 0.001) and sociotropic personality (*β* =  − 0.11, *t* (298) =  − 2.26, *p* < 0.05) demonstrated significance in predicting current suicidal ideation. Other simple slope analyses have shown that sociotropic effects on suicidality were significant when the level of TB was high (i.e., one standard deviation above the mean), *t* (294) =  − 2.62, *p* = 0.009), while autonomy was a predictor of suicide when PB levels were either low (i.e., one standard deviation below the mean), *t* (294) =  − 2.35, *p* = 0.019, or high (i.e., one standard deviation above the mean), *t* (294) = 2.56, *p* = 0.011, indicating autonomy to be the only risk factor for suicidality [60, 61]. Also, with regard to indirect effects, in a design with three-time points, depression symptoms seem to mediate the relationship between self-criticism and TB. A structural equation modelling showed that Time 1 autonomy predicted Time 2 depression symptoms (*β* = 0.137, *p* = 0.002), and Time 2 depression symptoms predicted Time 3 PB (*β* = 0.251, *p* = 0.002), as well as Time 3 TB (*β* = 0.283, *p* = 0.005) [56]. However, dependency, or neediness, was also shown to be significantly related to suicidality (*β* = 0.21, *t* = 4.71, *p* < 0.001; SE = 0.028, 95% CI [0.14, 0.30], *p* < 0.001) as well as self-criticism (*β* = 0.20, *t* = 4.17, *p* < 0.001; SE = 0.029, 95% CI [0.11, 0.30], *p* < 0.001) and depression (*β* = 0.57, *t* = 8.00, *p* < 0.001; SE = 0.004, 95% CI [0.44, 0.70], *p* < 0.001) indirectly through the effect of psychache and interpersonal needs (TB, PB) [22].

Distress, in terms of independent variable, also seems to indirectly mediate the relationship between personalities of self-criticism, dependency and suicidality dependent variables, with a mediational structural equation modelling (SEM) including self-criticism, dependency, distress and suicidality revealing that the relationship between self-criticism and high levels of suicidal behaviours was mediated by high levels of distress (*β* = 0.54, *t* = 6.452, *p* < 0.0001), as well as high levels of dependency significantly associated with high levels of distress (*β* = 0.36, *t* = 4.459, *p* < 0.0001), and high levels of distress significantly associated with suicidality (*β* = 0.51, *t* = 2.284, *p* < 0.022). Indirect association between high levels of self-criticism and high levels of suicidality (*z* = 2.18, *p* < 0.03) and high levels of dependency and high levels of suicidality (*z* = 2.08, *p* < 0.04) were also all found to be significant [23].

### Complicated grief

Higher scores of *interpersonal dependency* and *dependency on the spouse* have been found in chronic grievers ID: *M* = 0.31, SD = 0.88, F (4–80) = 3.30, *p* < 0.05; DOS: *M* = 0.19, SD = 0.86, F (4–80) = 2.58, *p* < 0.05) compared to resilient individuals (ID: *M* = 0.11, SD = 0.89, F (4–80) = 3.30, *p* < 0.05; DOS: *M* = 0.29, SD = 1.10, F (4–80) = 2.58, *p* < 0.05) [16]. Lower levels of *healthy dependency* have been found in prolonged grievers compared to resolved grievers MD = − 4.73, *p* = 0.015) [28], as well as lower levels of *healthy dependency* in prolonged grievers (*M* = 3.19, SD = 0.66, *F* = 5.16, *p* < 0.05) compared to resilient (*M* = 3.65, SD = 0.55, *F* = 5.16, *p* < 0.05) and recovered (*M* = 3.66, SD = 0.54, *F* = 5.16, *p* < 0.05) individuals, and higher levels of *destructive overdependence* in prolonged grievers (*M* = 2.85, SD = 0.83, *F* = 1.22, *p* < 0.25, DF = 2,62) compared to resilient individuals (*M* = 2.43, SD = 0.71, *F* = 1.22, *p* < 0.25), despite a little significance due to the small sample size [52]. A significant association between *individual’s promotion of dependency of the deceased* and grief score (*r *[129] = 21, *p* = 0.015) was also observed [65].

## Discussion

This systematic review was conducted in order to test the *Symptom Specificity Hypothesis* according to which anaclitic-sociotropic and introjective-autonomic personality dimensions are related to specific depression symptoms. More specifically, in line with this hypothesis, we hypothesised that a dependent-sociotropic-anaclitic personality style would have been related to more somatic symptoms and complaints such as crying, tearfulness, shame, loneliness, anger, anxiety symptoms, anhedonia and a more masked depressive form; while self-critical-autonomic-introjective personality would have been associated with cognitive symptoms, including failure feelings, self-hate, guilt, hostility, loss of interest and suicidality. Data collected showed a high heterogeneity and contrasting results across studies that do not totally support the hypothesis. In fact, most of the studies found weaker associations between somatic symptoms and dependent personalities. By contrast, as we had assumed, the relationship between self-criticism and cognitive symptomatology was significantly higher, with self-criticism being significantly associated with worthlessness, self-dislike, self-criticalness, defeat and failure, irritability, guilty feelings, self-hate, loss of interest, concentration difficulty, tiredness, changes in appetite and concerns about the ability to function [30, 47, 51, 69, 79]. Furthermore, self-criticism—in contrast to Blatt’s [12] view of the introjective configurations—seems to be able to predict poorer social functioning at follow-up [47], as well as both cognitive and somatic symptoms of depression [79]. Some studies, however, supported the symptoms specificity hypothesis, reporting a relationship between dependency and symptoms specifically associated with crying or tearfulness, loss and deprivation and helplessness [47], mood-variability, reactivity and loneliness [69], interpersonal awareness, separation anxiety, timidity, fragile inner self [59], and self-punitive symptoms [19].

Contrary to what we expected—according to the theoretical link between dependency and loneliness suggested by Blatt [12]—loneliness seems to be more closely related to the introjective personality than the anaclitic one, highlighting the interpersonal difficulties associated with the self-critical dimension [11, 55, 74, 90]. Also, an enhanced anhedonic symptomatology has been found to specifically characterise introjective individuals but not anaclitic individuals compared to normal ones [76]. In particular, Burke and Haslam [19] reported a link between anhedonia and core autonomy, a component of autonomy that comprised self-direction and freedom from attachments, this finding may account for the association between autonomy and endogenous depression reported by Peselow and colleagues [64]. Furthermore, self-emotions such as embarrassment [32] and guilt [19, 32] were shown to be associated both with sociotropy and autonomy, while shame appears to be the only emotion related to the introjective personality, supporting the opinion that shame is a fundamental emotion in the introjective personality, resulting in reduced interpersonal intimacy in these individuals [32, 75]. Finally, self-criticism also appears to be strongly associated with high levels of both state and trait anger, low anger control and high levels of anger towards the self and others, suggesting that introjective personality is characterised by hostile and irritable issues in depression [1]. The association between high levels of self-criticism and increased anger toward others could play an important role in explaining the associated vulnerability to depression. That is, the turning of anger towards others has been shown to lead to vicious interpersonal cycles characterised by increased feelings of frustration and anger in significant others, resulting in social exclusion and subsequent loneliness and depression [1, 11, 50, 74]. On the other hand, the anaclitic personality has been found to be associated with elevated levels of trait anger, low levels of anger directed toward others, and directing anger towards the self, suggesting that dependency is most closely related to depression associated with inhibited anger [1]. In this sense, some studies showed that dependent individuals often seem to underreport feelings of anger [1, 34, 73], indicating that they may fear that admitting anger towards others will lead to rejection and abandonment. Finally, the interaction between the introjective personality and depression symptoms has been found to predict insomnia, while no study identified the presence of sleep disorders in the anaclitic personality dimension.

Several studies [36, 37, 57, 71, 80], also tried to apply the Symptoms Specificity Hypothesis on suicidal behaviour in order to investigate whether different suicidal paths and patterns can be observed between the two groups, showing both sociotropy and autonomy to be associated with different suicidal characteristics. In particular, self-critical individuals indicate a greater intent to die, higher lethality behaviours, and higher risk and risk-rescue scores along with lower rescue scores compared to patients scoring lower in the introjective-autonomic personality, while sociotropics seem to show higher rescue scores and lower suicidal risk, lower intent to die and risk-rescue if compared to those lower in dependency and to self-critical individuals. Lower rescue scores in self-criticism show the tendency of these individuals to adopt more precautionary behaviours against the possibility of being discovered during the suicidal act compared to dependents and use more active practices of suicide such as firearms [8], highlighting the greater risk of these individuals for suicidality. Dependent people otherwise utilise fewer precautions against being discovered, by adopting less lethal and more passive suicide methods and attempts such as overdose [37]. In summary, self-critics and dependents are reported to have a different vulnerability to attempting suicide, even depending on interpersonal or intrapsychic life events [36]: while sociotropics are more worried about dependent issues, autonomic individuals show more suicidal thoughts and their suicidal acts seem to be gestures rather than attempts, showing a greater risk for successful suicide [80]. More specific differences have been found between younger and older adults in relation to three introjective-autonomic personality subcomponents, where in younger adults, suicidality appears to be associated with autonomy’s subscale of Perfectionism and Defensive Separation, in older adults only Need for Control autonomy’s subscale was related to increased propensity for suicidal behaviour. Data suggest that the autonomy personality and its propensity to suicide can be different in relation to suicide risk, with age having a mediation role. For this reason, suicidal behaviour could also differ across the life span [26], with the Need for Control subcomponent seeming to reflect inflexibility, which in turn is associated with suicidality in old age [27]. According to the *Interpersonal-Psychological Theory of Suicide* [44], four studies [60, 61], focused on the possibility that sociotropy and autonomy could contribute to the development of two interpersonal dysfunctions—PB and TB—that lead to suicide risk. Main results indicate an association between autonomy and PB and TB in predicting suicide, even with the mediation role of depression symptoms. Contrasting results, however, seem to show that sociotropy is also significantly related to suicidality through the indirect effect of PB and TB, and distress [23].

With regard to complicated grief, *DSM-5* defines this clinical condition as a chronic grief experience that follows the loss of a loved one and is frequently associated with the expression of various somatic complaints such as digestive problems and pain and fatigue, resembling in some cases the masked form of depression typical of the anaclitic personality dimension. For this reason, we hypothesised that dependency was a risk factor and a predictor of complicated grief.

## Conclusions

Overall, studies included in this review support the association between dependency and complicated grief, indirectly providing evidence of the hypothesised relationship between dependence and masked symptoms of depression. Nevertheless, it is important to note the limited number of studies examining the symptoms specificity hypothesis and their fragmented results, which in turn, leads to contrasting results in this review. This variability of results might be due to the fact that the selected studies do utilise different tests measuring anaclitic-sociotropic and introjective-autonomic personality styles because relatively little attention has been focused only on the original DEQ and SAS according to Blatt’s and Beck’s theories (1874, 1983). Particularly, these tests assess different subcomponents of these personalities. Thus, it would be useful to create more unitary methodologies of evaluation, combining the components measured by all the tests included in this review. In addition, many studies examining depression symptoms are based on diagnostic criteria including mainly cognitive symptoms rather than other depressive forms, such as the somatic one.

Future directions should provide data from experimental and longitudinal research to specifically investigate the symptoms specificity hypothesis and, thus, to corroborate the hypothesised correlation between personality styles and specific clusters of depression symptoms. This might make an important contribution to the clinical context in terms of therapeutic implications, supporting the existence of a form of depression characterised by somatic features which should not be ignored by the main diagnostic criteria currently in use. This would improve the implementation of more effective and personalised treatments built on the single individual and on different symptoms among depressed patients.

## Data Availability

The authors can confirm that all relevant data are included in the article and/or its Additional files.

## Appendix A

**Table:**

Study	Question described	Appropriate study design	Appropriate subject selection	Characteristics described	Random allocation	Researchers Blinded	Subject blinded	Outcome measures well defined and robust to bias		Sample size appropriate	Analytic methods well described	Estimate of variance reported	Controlled for confounding	Results reported in detail	Conclusion supported by results?	Rating (%)
Klein et al. [47]	2	2	1	1	NA	0	0	2		1	2	2	1	2	2	Moderate (64.3%)
Robins and Luten [69]	2	2	2	2	NA	0	0	1		1	2	1	1	2	2	Moderate (64.3%)
Robins et al. [71]	2	2	2	2	NA	0	0	1		2	2	0	2	2	2	Moderate (67.9%)
Desmet et al. [30]	2	2	2	2	NA	0	0	2		2	2	1	1	2	2	Moderate (71.4%)
Luyten et al. [51]	2	2	2	2	NA	0	0	2		1	2	2	2	2	2	Strong (75%)
Otani et al. [59]	2	2	1	2	NA	0	0	2		2	2	0	1	2	2	Moderate (64.3%)
Straccamore et al. [79]	2	2	2	2	NA	0	0	2		1	2	1	2	2	2	Moderate (71.4%)
Schachter and Zlotogorski [74]	2	2	1	1	NA	0	0	2		1	2	0	2	2	2	Moderate (60.7%)
Besser et al. [11]	2	2	1	2	NA	0	0	2		2	2	2	2	2	2	Strong (75%)
Dorahy and Hanna [32]	2	2	1	2	NA	0	0	2		2	2	2	2	2	2	Strong (75%)
Burke and Haslam [19]	2	2	2	2	NA	0	0	2		1	2	1	2	2	2	Moderate (71.4%)
Abi-Habib and Luyten [1]	2	2	1	2	NA	0	0	2		2	2	1	2	2	2	Moderate (71.4%)
Silva et al. [76]	2	2	2	2	NA	0	0	2		2	2	0	1	2	2	Moderate (67.9%)
Bar et al. [4]	2	2	1	2	NA	0	0	2		2	2	0	2	2	2	Moderate (67.9%)
Fazaa and Page [36]	2	2	1	2	NA	0	0	2		2	1	2	1	1	2	Moderate (64%)
Vanhuele and Desmet [80]	2	2	2	2	NA	0	0	2		2	1	2	1	1	2	Moderate (67.9%)
Faaza and Page [37]	2	2	1	2	NA	0	0	1		1	2	1	1	2	2	Moderate (60%)
O’Riley and Fiske [57]	2	2	1	2	NA	0	0	2		1	2	2	1	2	2	Moderate (67.8%)
Campos and Holden [21]	2	1	1	2	NA	0	0	2		2	2	2	1	2	2	Moderate (67.8%)
O’Keefe et al. [56]	2	2	1	2	NA	0	0	1		2	2	2	1	2	2	Moderate (67.8%)
Campos and Holden [22]	2	2	1	2	NA	0	0	1		2	2	2	1	2	2	Moderate (67.8%)
Park and Kim [60, 61]	2	2	1	2	NA	0	0	1		2	2	2	2	2	2	Moderate (71%)
Campos, Besser and Blatt [23]	2	1	1	2	NA	0	0	1		2	2	2	2	2	2	Moderate (67.8%)
Piper et al. [65]	2	2	2	2	NA	0	0	2		2	2	2	1	2	2	Strong (75%)
Bonanno et al. [16]	2	2	1	2	NA	0	0	1		2	2	1	2	2	2	Moderate (67.8%)
Denckla et al. [28]	2	2	1	1	NA	0	0	1		2	2	1	2	2	2	Moderate (64.2%)
Mancini et al. [52]	2	2	1	2	NA	0	0	1		2	2	1	2	2	2	Moderate (67.8%)

*NA* not applicable, 2 indicates *yes*, 1 indicates *partial,* 0 indicates *no*

Quality scores ≥ 75% strong, 56 ≥ 74% moderate, ≤ 55% weak

## Abbreviations

An: Anhedonia
Ang: Anger
BDI-II: Beck Depression Inventory-Second Edition
BSI: Brief symptom inventory
CDI: Clinical diagnostic interview
CES-D: Center for Epidemiologic Studies Depression scale
CG: Cognitive symptoms
CRSD: Carroll rating scale for depression
DABS: Dependency and Achievement Belief Scales
DEQ: Depressive Experiences Questionnaire
DES-IV: Differential Emotions Scale
DSI-SS: Depressive Symptoms Index Subscale
DSM-IV-TR: Diagnostic and Statistical Manual of Mental Disorders, fourth edition, text revised
DSM-5: Diagnostic and Statistical Manual of Mental Disorders, fifth edition
HAMD: Hamilton Depression Rating Scale
ICF: Inventory of clinical features
IDD: Inventory to Diagnose Depression
IDS: Interpersonal dependency scale
IES: Impact of events scale
In: Insomnia
INQ (TB–PB): Interpersonal Needs Questionnaire, thwarted belongingness and perceived burdensomeness subscales
INTREX: Structural Analysis of Social Behaviour Intrex Questionnaire
IPSM: Interpersonal sensitivity measure
ISI: Insomnia severity index
K-INQ14: Korean version of Interpersonal Needs Questionnaire
Lo: Loneliness
MCMI: Millon clinical multiaxial inventory
MDD: Major depressive disorder
PGI: Pathological grief items
PRISMA: Preferred reporting items for systematic reviews and meta-analyses
PSI: Personal Style Inventory
PSQI: Pittsburgh Sleep Quality Index
PTSD: Post-traumatic stress disorder
RPT: Relationship Profile Test
SADS: Schedule for affective disorders and Schizophrenia
SAS-SR: Social Adjustment Scale-Self-Report
SAS: Sociotropy/Autonomy Scale
SBQ-R: The Suicide Behaviours Questionnaire-Revised
SCID: Structured Clinical Interview for the DSM
SCL-90: Symptom Checklist-90
Sh: Shame
SI: Suicidal ideation
SIS: Suicide Intent Scale
SS: Somatic symptoms
STAXI: State–Trait Anger Expression Inventory
SRULS: UCLA Loneliness Scale-Revised
TRIG: Texas Revised Inventory of Grief
ZUNG-SDS: Zung Self-Rating Depression Scale
